# Real-world outcomes of immune checkpoint inhibitor-based combination therapy in older adult patients with metastatic renal cell carcinoma: a multi-center, retrospective analysis

**DOI:** 10.3389/fimmu.2025.1668406

**Published:** 2025-09-25

**Authors:** Hiroaki Ikoma, Shuzo Hamamoto, Yoshihiko Tasaki, Misato Tomita, Takuya Sakata, Hiroko Suzuki, Yusuke Noda, Masayuki Usami, Yohei Tsubouchi, Yoshihisa Mimura, Toshiharu Morikawa, Takashi Nagai, Rei Unno, Toshiki Etani, Taku Naiki, Yosuke Sugiyama, Takahiro Yasui

**Affiliations:** ^1^ Department of Nephro-Urology, Nagoya City University Graduate School of Medical Sciences, Nagoya, Japan; ^2^ Department of Clinical Pharmaceutics, Nagoya City University Graduate School of Medical Sciences, Nagoya, Japan; ^3^ Department of Urology, Kainan Hospital, Yatomi, Japan; ^4^ Department of Urology, Anjo Kosei Hospital, Anjo, Japan; ^5^ Department of Urology, Toyota Kosei Hospital, Toyota, Japan; ^6^ Department of Urology, Konan Kosei Hospital, Konan, Japan

**Keywords:** metastatic renal cell carcinoma, immune checkpoint inhibitor, tyrosine kinase inhibitor, older adult patient, adverse event

## Abstract

**Introduction:**

Immune checkpoint inhibitor (ICI)-based combination therapy has revolutionized first-line treatment outcomes for metastatic renal cell carcinoma (mRCC). In this study, we aimed to retrospectively analyze real-world clinical outcomes and toxicities of first-line ICI-based combination therapies, specifically nivolumab plus ipilimumab (IO+IO) and ICIs plus tyrosine kinase inhibitors (IO+TKI), in Japanese patients with mRCC aged ≥ 75 years compared with non-older adult patients.

**Methods:**

We retrospectively enrolled 156 patients with mRCC who received first-line IO+IO or IO+TKI between September 2018 and June 2024 at eight Japanese institutions. Patients were categorized into an older adult group (≥ 75 years, n=49) and a non-older adult group (< 75 years, n=107). We evaluated objective response rate (ORR), disease control rate (DCR), progression-free survival (PFS), overall survival (OS), and adverse events (AEs).

**Results:**

The overall ORR (47% vs. 59%, p=0.43) and DCR (86% vs. 83%, p=0.65) were comparable between groups. No significant differences were observed in PFS (median: 15.5 vs. 17.0 months, p=0.78) or OS (NA vs. 52.2 months, p=0.61). In the IO+IO regimen, the ORR, DCR, PFS, OS, and AE rates were comparable across age groups. However, in the IO+TKI regimen, the ORR was significantly lower in older adults (55% vs. 81%, p=0.04), and treatment discontinuation due to AEs was significantly higher in older adults (60% vs. 32%, p=0.02), with a shorter time to discontinuation despite no difference in the initial TKI dose and RDI. The non-older adult group showed significantly better PFS with IO+TKI compared with IO+IO (hazard ratio: 2.37, p=0.02). In contrast, in the older adult group, PFS and OS were approximately equivalent between the two regimens.

**Conclusion:**

Our real-world data indicated that ICI-based combination therapies are effective in patients with mRCC aged ≥75 years, with outcomes largely non-inferior to non-older adult patients. However, the comparable efficacy of IO+TKI and IO+IO in the older adult group, which may differs from that in the non-older adult group, highlights the importance of understanding the distinct characteristics of each regimen for individualized treatment selection and careful management, particularly regarding AE monitoring and dose adjustment in older adult patients receiving IO+TKI.

## 1 Introduction

Globally, population aging is progressing rapidly, with a proportion of individuals ≥ 65 years old expected to nearly double by 2050 (1). For example, in Japan, 29.3% of the population will be ≥ 65 years old, and 16.8% will be ≥ 75 years old by 2024 (2), making it one of the most aged societies in the world. The Japanese Cancer Registry estimated that approximately 29,000 new cases of renal cancer were diagnosed in 2020, with renal cell carcinoma (RCC) accounting for most. The proportion of patients ≥ 75 years old is 44.4% (3). Therefore, carefully considering treatment options for older adult patients with RCC is essential.

Immune checkpoint inhibitor (ICI)-based combination therapy has revolutionized first-line treatment outcomes by improving the survival and response rates of patients with metastatic RCC (mRCC) (4, 5). The CheckMate 214 trial demonstrated that nivolumab and ipilimumab combination therapy (IO+IO) showed superior efficacy compared with sunitinib (6, 7). Furthermore, the survival benefits of first-line ICI plus tyrosine kinase inhibitor (IO+TKI) therapies have been widely reported, and several regimens have been established as treatment options (8–11). However, the safety and efficacy of ICI-based combination therapies in older adult patients remain controversial. The proportion of older adult patients enrolled in clinical trials is relatively low, which limits the comprehensive evaluation of treatment safety and efficacy. This is primarily due to older adult patients often meeting the exclusion criteria, including multiple primary cancers, impaired renal function, or reduced cardiac function. For example, patients aged ≥ 70 years accounted for only 17.4% of the total population in the CheckMate 214 trial (6), and in the KEYNOTE-426 trial, patients aged ≥ 65 years comprised approximately 40% of the total population (9). There have been reports that the average age of patients with RCC in real-world settings is 6.49 years older than those included in clinical trials (12), indicating a discrepancy between real-world and clinical trial data regarding patient age. Furthermore, immune aging is characterized by impaired T cell function and altered inflammatory environments, potentially causing reduced ICI effectiveness (13, 14). However, it is unclear whether these treatment strategies offer comparable efficacy and safety in older adult and non-older adult patients.

Therefore, evaluating the treatment outcomes in older adult patients and comparing them with those of non-older adult patients is crucial. In this study, we aimed to retrospectively analyze the clinical outcomes and toxicities of IO+IO and IO+TKI as first-line combination therapies in Japanese patients with mRCC aged ≥ 75 years.

## 2 Materials and methods

### 2.1 Patients and methods

The Ethics Review Board of the Nagoya City University Graduate School of Medical Sciences approved this study (Approval Number: 60-19-0196), and it was conducted in accordance with the guidelines of the Declaration of Helsinki. In this retrospective study, we enrolled 156 patients diagnosed with mRCC who received first-line IO+IO or IO+TKI therapy between September 2018 and June 2024 at Nagoya City University Hospital and seven affiliated institutions. We classified patients aged ≥ 75 years as the older adult group and those aged < 75 years as the non-older adult group using a previous report showing that the global median age at diagnosis of kidney cancer is 75 years (15). RCC diagnosis was confirmed by experienced pathologists through histological analysis. The choice of ICI-based combination therapy was made after discussion between the attending physician and other urologists, and after considering patient characteristics using the International Metastatic Renal Cell Carcinoma Database Consortium (IMDC) risk classification. All favorable-risk patients were assigned to IO+TKI therapy. Informed consent was obtained from all patients, and treatment regimens were determined with their full agreement. Baseline and on-treatment assessments included medical history, demographic and physical examinations, Karnofsky Performance Status (KPS), age-unadjusted Charlson Comorbidity Index (CCI) (16), and blood and urine tests, all conducted at the discretion of the attending physician. Treatment response after initiating ICI-based combination therapy was assessed according to the Response Evaluation Criteria in Solid Tumors version 1.1 (17) at physician-scheduled intervals. Toxicity was graded using the Common Terminology Criteria for Adverse Events version 5.0. Adverse events (AEs) occurring within the first two months after treatment initiation were specifically used in the analysis. Blood test values were obtained on the day before or the day of treatment initiation. The dosage and duration of TKI administration were assessed in all the patients undergoing IO+TKI treatment. Relative dose intensity (RDI) was calculated as the ratio of the actual administered dose to the planned maximum dose from treatment initiation to discontinuation.

### 2.2 Statistical analysis

The chi-square test or Fisher’s exact test (for stratification factors) and the Mann–Whitney U test (for continuous variables) were used to compare patient characteristics. In addition, the interquartile range (IQR) was used to report continuous variables. progression-free survival (PFS) and overall survival (OS) were stratified using the Kaplan–Meier method and analyzed using the log-rank test. The Cox proportional hazards model was used to analyze the hazard ratios (HR) and 95% confidence intervals (CI). All reported p-values were two-sided, with statistical significance set at p < 0.05. Statistical analyses were performed using the EZR software (Saitama Medical Center, Jichi Medical University, Saitama, Japan) (18). Patients with favorable IMDC risk were excluded to ensure balanced baseline conditions and to compare the treatment outcomes between the IO+IO and IO+TKI groups.

## 3 Results

### 3.1 Patient characteristics

**Table 1** presents the baseline characteristics of patients before the first course of ICI-based combination therapy, categorized from the total (n = 156) into the older adult group (n = 49, 31.4%) and the non-older adult group (n = 107, 68.6%). The median follow-up period following initiation of the ICI-based combination therapy was 13.9 and 25.9 months (IQR: 6.5–32.2 and 8.1–34.3 months, respectively) in the older adult and non-older adult groups (p=0.19). In the older adult group, 24 patients (49%) received IO+IO, and 25 patients (51%) received IO+TKI. In the non-older adult group, 66 and 41 patients (62 and 38%) received IO+IO and IO+TKI, respectively. There was no significant difference between the groups (p=0.14). Details of each IO+TKI regimen are provided in **Supplementary Table S1**.

**Table 1:** Patient characteristics.

	Total (n=156)	Older adult (n=49)	Non-older adult (n=107)	P value
Age (years) median [IQR]	71 [64-76]	79 [77-82]	68 [57-71]	<0.01
Gender: Male, n (%)	118 (76)	34 (69)	84 (79)	0.21
BMI, kg/m^2^ median [IQR]	22.3 [19.5-24.6]	20.7 [19.0-23.6]	22.7 [19.8-24.7]	0.09
KPS: ≥80, n (%)	126 (81)	40 (82)	86 (80)	0.85
Charlson comorbidity index (CCI)				0.25
0	75 (48)	21 (43)	54 (51)	
1-2	69 (44)	25 (51)	44 (41)	
≥ 3	12 (8)	3 (6)	9 (8)	
Baseline corticosteroid use: Yes	5 (3)	2 (4)	3 (3)	0.65
Histopathology, n (%)				
Clear cell	119 (76)	37 (76)	82 (77)	0.55
Others	27 (17)	10 (20)	17 (16)	
unknown	10 (7)	2 (4)	8 (7)	
Prior nephrectomy: Yes, n (%)	67 (43)	16 (33)	51 (48)	0.08
Number of metastasis site, n (%)				
1	77 (49)	24 (49)	53 (50)	0.92
2	50 (32)	15 (31)	35 (33)	
≥3	29 (19)	10 (20)	19 (18)	
Metastasis site, n (%)				
Lung: Yes	107 (69)	31 (63)	76 (71)	0.33
Liver: Yes	18 (11)	6 (12)	12 (11)	0.85
Bone: Yes	45 (29)	10 (20)	35 (33)	0.12
IMDC risk classification, n (%)				
favorable	10 (6)	2 (4)	8 (7)	0.58
Intermediate	74 (48)	26 (53)	49 (46)	
Poor	72 (46)	21 (43)	50 (47)	
Blood test, n (%)				
CRP≥1.0	80 (51)	25 (51)	55 (51)	0.96
NLR>2.8	102 (65)	29 (59)	73 (68)	0.27
PLR>215.6	58 (37)	10 (20)	48 (45)	<0.01
Anemia: Yes	97 (62)	35 (71)	62 (58)	0.10
Hi-neutrophil: Yes	19 (12)	4 (8)	15 (14)	0.30
Combination Therapy, n (%)				0.14
IO+IO	90 (58)	24 (49)	66 (62)	
IO+TKI	66 (42)	25 (51)	41 (38)	

IQR, interquartile range; BMI, Body mass index; KPS, Karnofsky performance status; IMDC, International Metastatic Renal Cell Carcinoma Database Consortium; CRP, C-reactive protein; NLR, neutrophil-to-lymphocyte ratio; PLR, platelet-to-lymphocyte ratio

### 3.2 Antitumor efficacy

**Table 2** presents the objective response rate (ORR), disease control rate (DCR), and best treatment response for each group. The ORR and DCR were comparable between both groups (ORR: 47% vs. 59%, p=0.43; DCR: 86% vs. 83%, p=0.65). The best responses were not significantly different between the two groups (p=0.10).

**Table 2:** Treatment outcomes.

	All regimen				IO+IO				IO+TKI			
	Total	Older adult	Non-older adult	p-value	Total	Older adult	Non-older adult	p-value	Total	Older adult	Non-older adult	p-value
	(n=156)	(n=49)	(n=107)		(n=90)	(n=24)	(n=66)		(n=66)	(n=25)	(n=41)	
Objective response, n (%)	79 (51)	20 (41)	59 (55)	0.01	38 (42)	9 (38)	29 (44)	0.57	41 (62)	11 (44)	30 (73)	0.02
Disease control, n (%)	120 (77)	37 (76)	83 (78)	0.78	65 (72)	18 (75)	47 (71)	0.73	55 (83)	19 (76)	36 (88)	0.21
Best response, n (%)				0.10				0.69				0.07
emspCR	14 (9)	5 (10)	9 (9)		8 (8)	2 (4)	6 (5)		6 (9)	3 (12)	3 (7)	
emspPR	65 (42)	15 (31)	50 (47)		30 (33)	7 (29)	23 (39)		35 (53)	8 (32)	27 (66)	
emspSD	41 (26)	17 (35)	24 (22)		27 (30)	9 (38)	18 (27)		14 (21)	8 (32)	6 (15)	
emspPD	23 (15)	6 (12)	17 (15)		21 (24)	5 (21)	16 (24)		2 (3)	1 (4)	1 (2)	
emspNot assesed	13 (8)	6 (12)	7 (7)		4 (4)	1 (4)	3 (5)		9 (14)	5 (20)	4 (10)	

CR, Complete Response; PR, Partial Response; SD, Stable Disease; PD, Progressive Disease.

We compared IO+IO and IO+TKI separately and analyzed the data. In the IO+IO regimen, ORR (39% vs. 46%, p=0.57) and DCR (76% vs. 78%, p=0.73) did not differ significantly between the two regimens. In contrast, the ORR (55% vs. 81%, p=0.04) in the IO+TKI regimen was significantly lower in the older adult group. In the older adult group, the response rates of IO+IO and IO+TKI were comparable (ORR: 39% vs. 55%, p=0.30; DCR: 78% vs. 95%, p=0.11).

**Figure 1:**
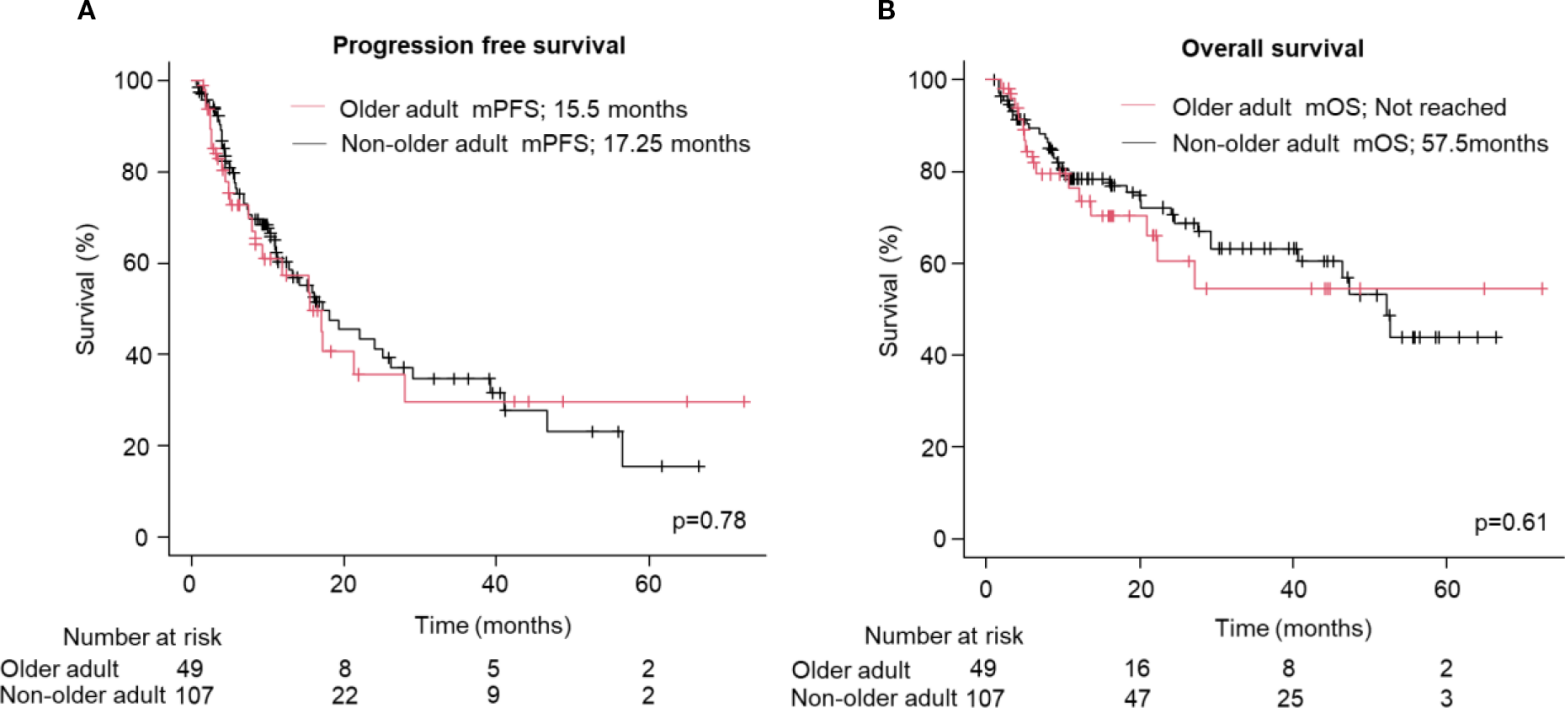
Progression free and overall survival following treatment with ICI-based combination therapy. **(A, B)** Kaplan-Meier survival curves for **(A)** progression free survival (Older adult group: n = 49; Non-older adult group: n = 107) and **(B)** overall survival (Older adult group: n = 49; Non-older adult group: n = 107) in patients. **(A, B)** Log-rank test. mPFS, median progression free survival; mOS, median overall survival.

**Figure 2:**
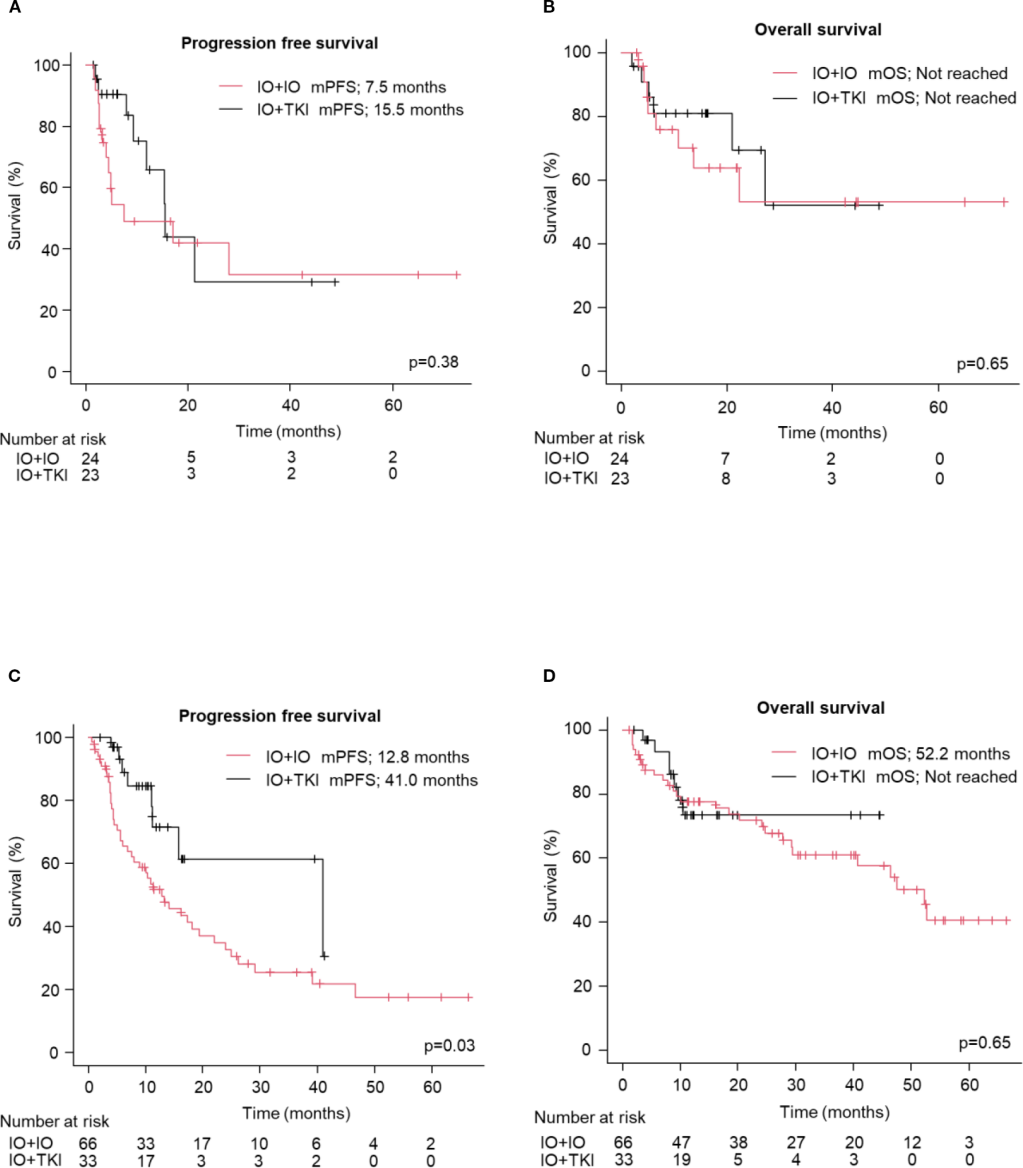
Progression free and overall survival following treatment with ICI-based combination therapy (except for IMDC: favorable). **(A, B)** Kaplan-Meier survival curves in the older adult group for **(A)** progression free survival (IO+IO: n = 24; IO+TKI: n = 23) and **(B)** overall survival (IO+IO: n = 24; IO+TKI: n = 23) in patients. **(C, D)** Kaplan-Meier survival curves in the Non-older adult group for **(C)** progression free survival (IO+IO: n = 66; IO+TKI: n = 33) and **(D)** overall survival (IO+IO: n = 66; IO+TKI: n = 33) in patients. **(A–D)** Log-rank test. mPFS, median progression free survival; mOS, median overall survival; ICI, immune checkpoint inhibitors; TKI, tyrosine kinase.

No significant differences in PFS (median: 15.5 vs. 17.0 months; HR: 1.07; 95% CI: 0.66–1.75, p=0.78, **Figure 1A**) and OS (Not reached vs. 52.2 months; HR: 1.17; 95% CI: 0.64–2.16, p=0.61, **Figure 1B**) were observed between the older adult and the non-older adult groups. The comparison of the two groups, excluding favorable-risk patients, is shown in **Supplementary Figure S1** which also presents no significant differences between two groups. PFS and OS were comparable between the two groups when stratified by the treatment regimen (**Supplementary Figure S2**). **Figure 2** presents the results of the subgroup analysis. In the older adult group, there was no significant difference in PFS (HR: 1.49; 95% CI: 0.61–3.64, p=0.38, **Figure 2A**) or OS (HR: 1.14; 95% CI: 0.41–3.16, p=0.65, **Figure 2B**) between the IO+IO and IO+TKI regimens. In contrast, in the subgroup analysis of the non-older adult group, PFS was significantly better in the IO+TKI regimen (HR: 2.37; 95% CI: 1.11–5.10, p=0.03, **Figure 2C**); however, no significant difference was observed in OS (HR: 1.23; 95% CI: 0.52–2.90, p=0.65, **Figure 2D**).

### 3.3 Adverse events

**Figure 3:**
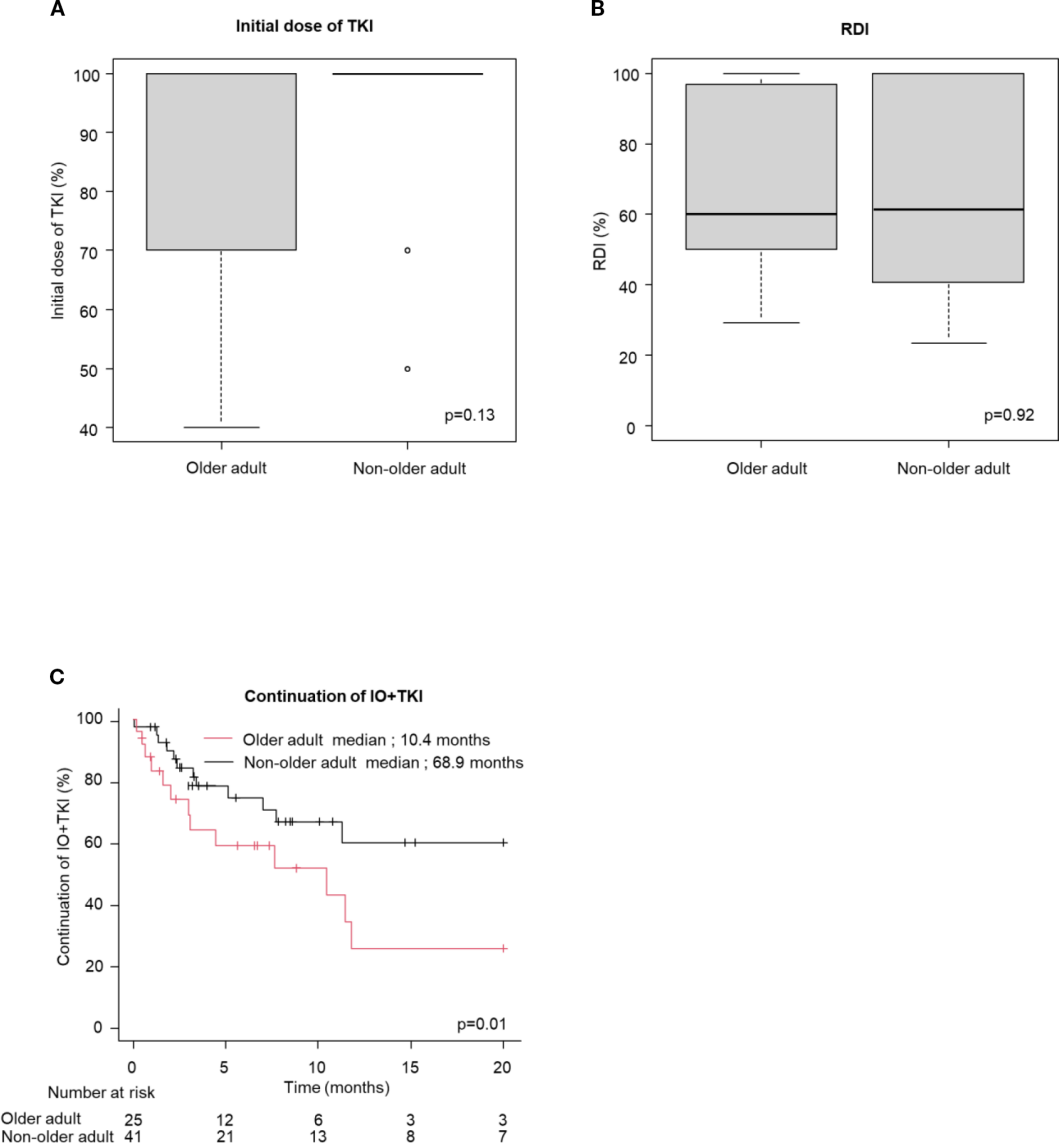
Initial dose of TKI and RDI, the time from initiating the first therapy to discontinuation owing to AEs (continuation of IO+TKI) **(A, B)** box plot for **(A)** initial dose of TKI (Older adult group: n = 22; Non-older adult group: n = 34) and **(B)** RDI (Older adult group: n = 25; Non-older adult group: n = 41), **(C)** Kaplan-Meier survival curves for continuation of IO+TKI (older adult group: n = 25; Non-older adult group: n = 41) in IO+TKI. **(A, B)** Mann-Whitney U test. **(C)** Log-rank test.

**Table 3** shows the frequencies of AEs. AEs occurred in 76% and 74% of the older adult and non-older adult groups, respectively (p=0.82). Grade ≥ 3 AEs occurred in 39% vs. 35%. (p=0.61). No significant differences were observed in treatment discontinuation due to AEs (25% vs. 20%, p=0.59) or steroid administration (29 vs. 30%, p=0.92) between the older adult and non-older adult groups in the IO+IO regimen. In contrast, older adult patients in the IO+TKI regimen had significantly higher treatment discontinuation rates owing to AEs (60% vs. 32%, p=0.02). **Figure 3** presents a comparison between the initial TKI dose and RDI, the duration of IO+TKI administration. No significant difference was observed in the initial TKI dose (p=0.13, **Figure 3A**) and RDI (p=0.92, **Figure 3B**) between the two groups. However, the time from initiating the first therapy to discontinuation due to AEs was significantly shorter in the older adult group (median: 10.4 vs. 68.9 months; HR: 2.53; 95% CI: 1.18–5.43, p=0.78, **Figure 3C**). Among the 15 older adult patients who discontinued treatment, 12 (80%) experienced multiple AEs. The detailed AEs profiles are summarized in **Supplementary Table S2**.

**Table 3:** Summary of AEs.

	All regimen				IO+IO				IO+TKI			
	Total	Older adult	Non-older adult	p-value	Total	Older adult	Non-older adult	p-value	Total	Older adult	Non-older adult	p-value
	(n=156)	(n=49)	(n=107)		(n=90)	(n=24)	(n=66)		(n=66)	(n=25)	(n=41)	
Any grades, n (%)	116 (66)	37 (76)	79 (74)	0.82	59 (66)	17 (71)	42 (64)	0.53	57 (86)	20 (80)	37 (90)	0.24
Grade 3 or more, n (%)	56 (41)	19 (39)	37 (35)	0.61	37 (41)	11 (46)	26 (39)	0.58	19 (29)	8 (32)	11 (27)	0.65
Discontinuation due to AE, n (%)	47 (30)	21 (43)	26 (24)	0.02	19 (21)	6 (25)	13 (20)	0.59	28 (42)	15 (60)	13 (32)	0.02

AE, adverse event; PSL, prednisolone.

**Figure 4:**
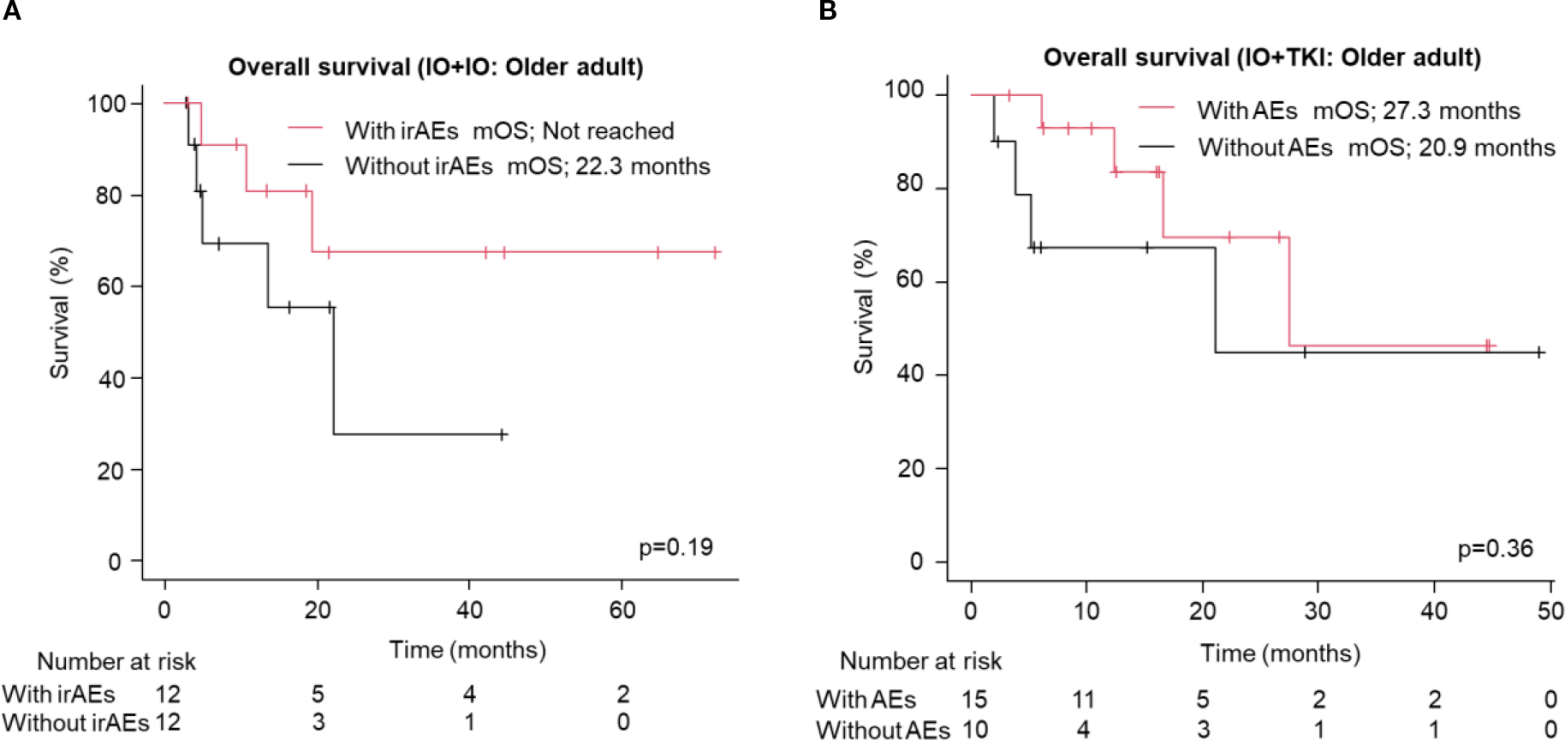
Overall survival in the older adult group following treatment with ICI-based combination therapies. **(A, B)** Kaplan-Meier survival curves for **(A)** IO+IO (With irAEs: N = 12; Without irAEs: N = 12) and **(B)** IO+TKI (With AEs: N = 15; Without AEs: N = 10) in patients. AEs, adverse events.

We analyzed the association between early AEs, occurring within the first two months of treatment, and OS for each regimen. The presence of early immune-related AEs (irAEs) or AEs was associated with longer OS in the IO+IO regimen (HR: 0.41, 95% CI: 0.20–0.81, p=0.01, **Supplementary Figure S3A**), but no significant association was found in the IO+TKI regimen (HR: 0.64, 95% CI: 0.24-1.72, p=0.38, **Supplementary Figure S3B**). A similar trend was observed in older patients, although the association did not reach statistical significance in either the IO+IO (HR: 0.39, 95% CI: 0.09–1.67, p=0.19, **Figure 4A**) or IO+TKI groups (HR: 0.52, 95% CI: 0.13–2.10, p=0.36, **Figure 4B**).

## 4 Discussion

In this study, we revealed no significant differences in the PFS, OS, or AEs frequency between the older adult and non-older adult groups. Our findings indicated that ICI-based combination therapies, when managed appropriately, can be effective in older adult patients with mRCC. We propose that treatment decisions should not be based solely on chronological age; rather, the characteristics of each regimen should be considered.

Focusing on IO+IO, we found similar response rates (ORR and DCR) across both age groups. Crucially, the incidence and severity of irAEs, alongside the rates of treatment discontinuation owing to irAEs, were comparable between the older adults and non-older adult groups.

These real-world observations regarding IO+IO in older adults are consistent with a growing body of evidence. The CheckMate 214 trial’s age-stratified analysis of OS did not show the superiority of IO+IO over sunitinib in older adult patients definitively (6); however, its conditional OS was favorable even in this group, suggesting its contribution to long-term outcomes (7). Furthermore, real-world data from the IMDC stated that survival benefits from ICI-based combination therapies are maintained even in patients aged ≥ 70 years, emphasizing that only chronological age should not dictate treatment selection (19). In addition, several studies have demonstrated non-inferior IO+IO treatment outcomes in older adult patients compared with younger patients, reinforcing our findings (20–23).

In the IO+TKI cohort, the older adult group showed a lower ORR trend than the non-older adult group. Nevertheless, the DOR was nearly identical, and no significant differences were observed in OS or PFS. The overall incidence of AEs was similar between groups; however, treatment discontinuation due to AEs was significantly higher. Moreover, the time to discontinuation was shorter in older adults, despite no differences in the initial TKI dose or RDI. These findings suggest that, in the IO+TKI regimen, older adults may discontinue treatment earlier due to AEs even when receiving the same initial TKI dose as non-older adults, despite experiencing a similar overall frequency of AEs. Therefore, a greater reduction in the TKI dose than in non-older adults may help maintain treatment in older adults. Several subgroup analyses have been conducted in clinical trials on IO+TKI therapy. Tomita et al. suggested that patients > 75 years old who underwent avelumab plus axitinib treatment would have a similar survival benefit as those aged 65–74 years (24). Varkaris et al. reported that treatment comprising pembrolizumab and axitinib prolonged PFS and OS compared with sunitinib even in patients aged ≥ 65 years (25). While there are a few real-world reports, Iinuma et al. reported that the median PFS for patients aged ≥ 70 years was significantly shorter than for patients aged < 70 years in their study utilizing various IO+TKI regimens (26). However, these findings were based on a relatively small cohort of 51 patients. More studies are needed to clarify the optimal management and outcomes of IO+TKI therapy in older adults.

Kaymakcalan et al. and Donskov et al. identified older age as a predictive factor for treatment discontinuation due to AEs among patients treated with TKI monotherapies (27, 28). However, Carina et al. reported that the increased incidence of treatment interruptions and dose reductions among older adult patients did not affect the overall efficacy despite the significantly lower TKI doses observed. Sustained drug exposure in each patient, rather than the absolute dose in milligrams, contributes to the clinical benefit of targeted therapies (29). These studies focused on TKI monotherapy; however, their findings share commonalities with our results in IO+TKI combination therapy, highlighting the importance of dose management, including TKI dose reduction. A recent report proposed the “start-low, go-slow” (SLGS) strategy, which involves initiating cancer treatment at lower-than-standard doses in older patients with solid tumors to reduce toxicity without affecting survival (30). Our findings suggest that in IO+TKI treatment of mRCC, SLGS strategy may help mitigate toxicity and prolong the duration of first-line therapy.

In previous reports, the occurrence of irAEs or treatment discontinuation due to irAEs did not negatively impact OS. The early occurrence of irAEs has been suggested to reflect immune activation, and in some cases, may even prolong OS (31–33). In our study, the results for the IO+IO regimen support this finding. Although the data for older patients were limited and the difference was not statistically significant, the presence of irAEs suggested a potential benefit to OS. In contrast, the lack of a significant association in the IO+TKI regimen may be due to the high number of TKI-related AEs. These results highlight the clinical significance of AEs and the need for further investigation.

Previous studies have shown that while IO+TKI therapy offers superior PFS compared with IO+IO, OS remains similar (34–36). The favorable PFS of IO+TKI is thought to result from the interaction between tumor immunity and angiogenesis, as anti-angiogenic therapy may enhance ICI efficacy (37). However, while IO+IO demonstrates inferior PFS compared with IO+TKI, it is considered non-inferior in OS due to several factors: the potential for durable long-term responses and better treatment-free survival (38), the sustained efficacy of vascular endothelial growth factor-TKIs even after ICI-based combination therapy (39, 40), and the fact that PFS is not a perfect surrogate for OS (41). Our study showed similar results in the non-older adult cohort; however, we suggest that this trend may be attenuated in older adults. In addition, the attenuated trend may be attributable to the higher frequency of treatment discontinuation due to AEs with IO+TKI therapy in the older adult group. These findings may have important implications for treatment decision-making in older adults. However, the small sample size limits the statistical power of this analysis, and the results should be interpreted with caution.

This study has some limitations. First, the participants were few, and this was a retrospective study. While the number of patients is especially limited for each IO+TKI regimen, real-world data on IO+TKI therapy in older adult patients remain scarce, underscoring the significance of our findings. Second, there were no explicit criteria for regimen selection, and the choice was left to the treating physician’s discretion. Particularly, the selection of IO+TKI regimens lacked uniformity. Such biases inherent in retrospective, real-world studies were recognized in other analyses concerning IO+IO by our group (42, 43). Third, data regarding geriatric assessment were insufficient, so the KPS and CCI were used as surrogate indicators. Fourth, the participants in our study with mRCC were all Japanese, and the clinical outcomes of ICI combination therapy could have been influenced by geographic region and ethnicity. Therefore, patient bias could not be controlled. Larger-scale studies are warranted.

In conclusion, our findings suggest that the treatment outcomes of immune combination therapy in patients with mRCC aged ≥ 75 years are not inferior to those in patients younger than 75. IO+IO and IO+TKI are distinct treatment modalities; therefore, understanding their individual characteristics is crucial for appropriate treatment selection and management. ICI-based combination therapy may be an effective treatment option, even in older adult patients.

## Data Availability

The raw data supporting the conclusions of this article will be made available by the authors, without undue reservation.
