# Supplementary material for: Real-world outcomes of immune checkpoint inhibitor-based combination therapy in older adult patients with metastatic renal cell carcinoma: a multi-center, retrospective analysis
Source: Front Immunol. 2025 Sep 25;16:1668406. doi: 10.3389/fimmu.2025.1668406 (PMC12507601; doi:10.3389/fimmu.2025.1668406)
Supplement: Supplementary file 1 [file DataSheet1.docx]

Supplementary Material

**Supplementary Tables**

**Table S1. Details of the IO + TKI regimen**

|  | Total (n=66) | Older adult (n=25) | Non-older adult (n=41) | p-value |
| --- | --- | --- | --- | --- |
| Regimen of IO+TKI | | | | 0.54 |
| Avelumab+Axitinib | 7 (11) | 4 (16) | 3 (7) |  |
| Pembrolizumab+Axitinib | 9 (14) | 3 (12) | 6 (15) |  |
| Nivolumab+Cabozantinib | 12 (18) | 5 (20) | 7 (17) |  |
| Pembrolizumab+Lemvatinib | 38 (58) | 13 (52) | 25 (61) |  |

**Table S2. Details of the AEs profiles**

|  | Older adult (n=49) | | Non-older adult (n=107) | |
| --- | --- | --- | --- | --- |
| Profile of AEs, n, (%) | Any Grade | Grade 3 or more | Any Grade | Grade 3 or more |
| All event | 37 (76) | 19 (39) | 79 (74) | 37 (35) |
| Dermatitis or Pruritus | 13 (27) | 3 (6) | 26 (24) | 2 (2) |
| Hypothyroidism | 9 (18) | 1 (2) | 19 (18) | 2 (2) |
| Trouble of intestine | 7 (14) | 3 (6) | 18 (17) | 5 (5) |
| Adrenal insufficiency | 6 (12) | 2 (4) | 7 (7) | 4 (4) |
| Hepatitis | 6 (12) | 1 (2) | 13 (12) | 7 (7) |
| Hypertension | 5 (10) | 1 (2) | 17 (16) | 3 (3) |
| Hand and foot syndrome | 5 (10) | 1 (2) | 9 (8) | 0 (0) |
| Type I DM | 3 (6) | 2 (4) | 4 (4) | 3 (3) |
| Interstitial pneumonia | 3 (6) | 1 (2) | 8 (7) | 5 (5) |
| Pituitary dysfunction | 2 (4) | 2 (4) | 3 (3) | 3 (3) |
| Proteinuria | 2 (4) | 1 (2) | 3 (3) | 0 (0) |
| Hoarseness | 2 (4) | 0 (0) | 3 (3) | 0 (0) |
| Neuropathy | 1 (2) | 1 (2) | 6 (6) | 2 (2) |
| Myocarditis | 1 (2) | 1 (2) | 3 (3) | 3 (3) |
| Kidney dysfunction | 1 (2) | 1 (2) | 1 (1) | 1 (1) |
| Destructive thyroiditis | 1 (2) | 0 (0) | 7 (7) | 3 (3) |
| Stomatitis | 1 (2) | 0 (0) | 2 (2) | 0 (0) |
| Myasthenia gravis | 1 (2) | 0 (0) | 1 (1) | 1 (1) |
| Encephalitis | 0 (0) | 0 (0) | 1 (1) | 1 (1) |

Abbreviations: AEs, adverse events; DM, diabetes mellitus

**Supplementary Figures**

**Figure S1.** Progression free and Overall survival following treatment with ICI-based combination therapy; excluding favorable-risk. (A, B) Kaplan-Meier survival curves for (A) progression free survival (Older adult group: n=47; Non-older adult group: n=99; hazard ratio [HR]: 0.99, 95% confidence interval [CI]: 0.60–1.65, p=0.97) and (B) overall survival (Older adult group: n=47; Non-older adult group: n=99; HR: 1.10, 95% CI: 0.59–2.07, p=0.76) in patients. (A, B) Log-rank test. mPFS, median progression free survival; mOS, median overall survival.

**Figure S2.** Progression free and Overall survival following treatment ICI-based combination therapies. (A, B) Kaplan-Meier survival curves (IO+IO) for (A) progression free survival (Older adult group: n=24; Non-older adult group: n=66; HR: 0.98, 95% CI: 0.52–1.82, p=0.94) and (B) overall survival (Older adult group: n=24; Non-older adult group: n=66; HR: 1.09, 95% CI: 0.49–2.43, p=0.83) in patients. (C, D) Kaplan-Meier survival curves (IO+TKI) for (C) progression free survival (Older adult group: n=25; Non-older adult group: n=41; HR: 1.85, 95% CI: 0.75–4.56, p=0.18) and (D) overall survival (Older adult group: n=25; Non-older adult group: n=41; HR: 1.38, 95% CI: 0.50–3.82, p=0.54) in patients. (A-D) Log-rank test. mPFS, median progression free survival; mOS, median overall survival; ICI, immune checkpoint inhibitors; TKI, tyrosine kinase.

**Figure S3.** Overall survival in total following treatment with ICI-based combination therapies. (A, B) Kaplan-Meier survival curves for (A) IO+IO (With irAEs: n=44; Without irAEs: n=46) and (B) IO+TKI (With AEs: n=28; Without AEs: n=38) in patients. (A, B) Log-rank test. mPFS, median progression free survival; mOS, median overall survival, AEs, adverse events.
