# Supplementary figures and images for: Real-world outcomes of immune checkpoint inhibitor-based combination therapy in older adult patients with metastatic renal cell carcinoma: a multi-center, retrospective analysis
Source: Front Immunol. 2025 Sep 25;16:1668406. doi: 10.3389/fimmu.2025.1668406 (PMC12507601; doi:10.3389/fimmu.2025.1668406)

FigureS1

A

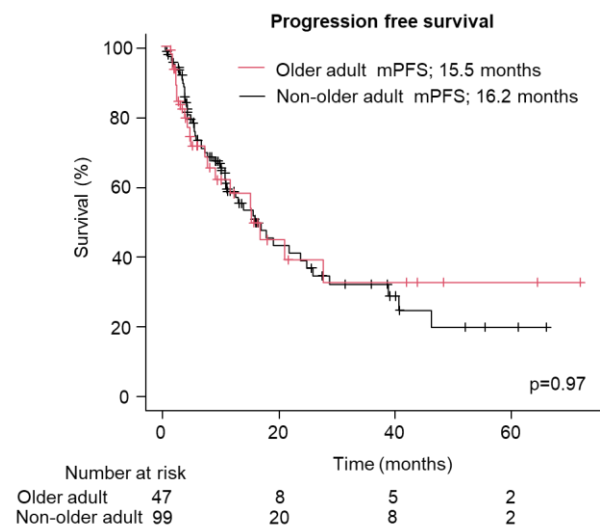

B

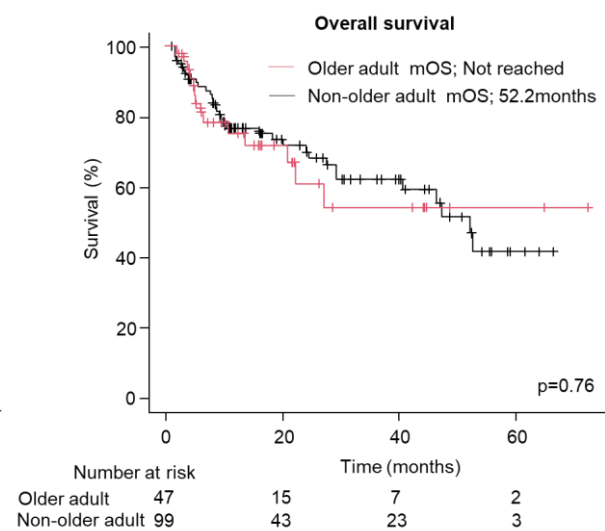

FigureS2

A

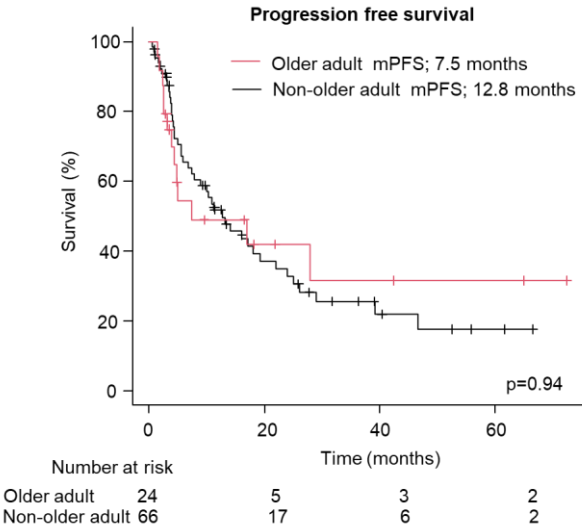

B

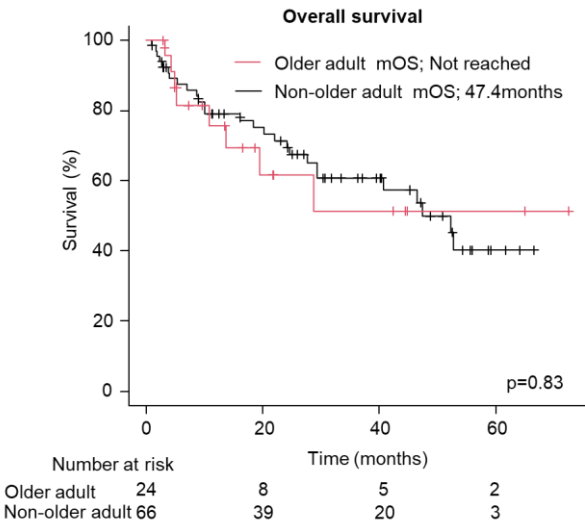

C

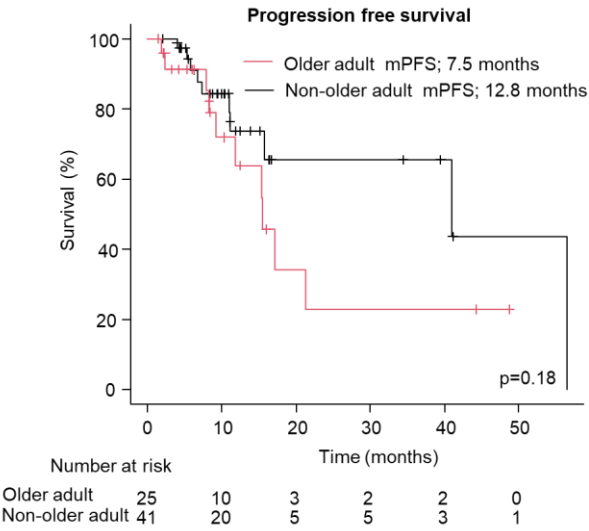

D

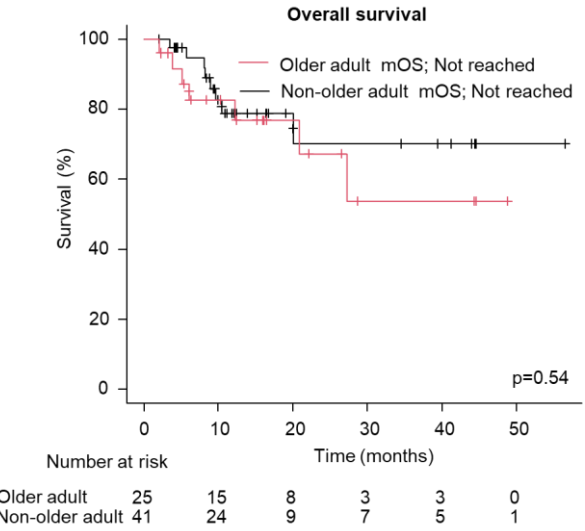

FigureS3

A

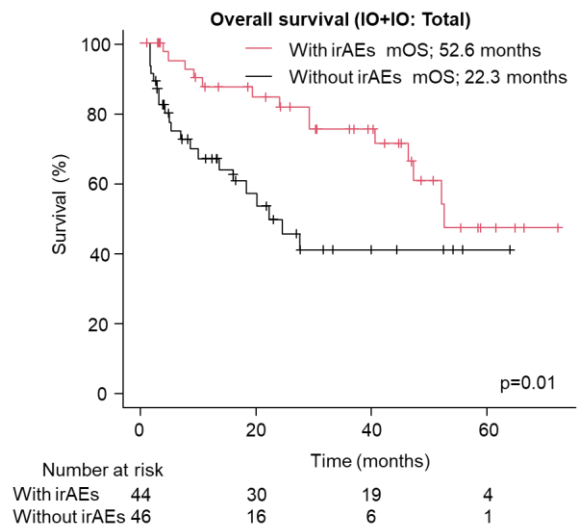

B

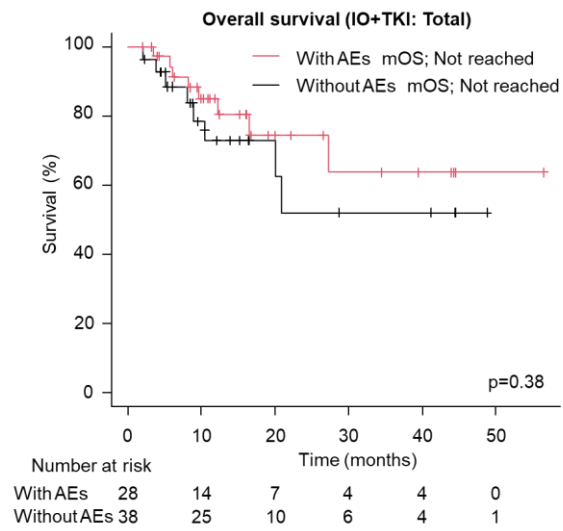

Supplement: Supplementary file 2 [file DataSheet2.pdf]
